# Gas engine CCHP system optimization: An energy, exergy, economic, and environment analysis and optimization based on developed northern goshawk optimization algorithm

**DOI:** 10.1016/j.heliyon.2024.e31208

**Published:** 2024-05-14

**Authors:** Jiangping Nan, Qi Xiao, Milad Teimourian

**Affiliations:** a Xi'an Traffic Engineering Institute, Xi'an, 710300, Shaanxi, China; b CCTEG Xi'an Research Institute, Xi'an, 710076, Shaanxi, China; c Young Researchers and Elite Club, Ardabil Branch, Islamic Azad University, Ardabil, Iran; d College of Technical Engineering, The Islamic University, Najaf, Iraq

**Keywords:** Energy analysis, Exergy analysis, Economic assessment, Environmental impact assessment, System sizing, Gas engine system, Combined cooling heating and power (CCHP), Developed northern goshawk optimization

## Abstract

This paper aims to enhance the design and operation of a Combined Cooling, Heating, and Power (CCHP) system utilizing a gas engine as the primary energy source for a residential building in China. An Energy, Exergy, Economic, and Environment (4E) analysis is employed to assess the system's performance and impact based on energy, exergy, economic, and environmental criteria. The effectiveness of the DNGO algorithm is evaluated on a case study site and compared with Northern Goshawk Optimization (NGO) and Genetic Algorithm (GA). The findings demonstrate that the DNGO algorithm identifies the optimal gas engine size of 130 kW. The algorithm's search capabilities are greatly enhanced by this unique blend, surpassing what traditional methods can offer. The DNGO algorithm brings several advantages, including unparalleled energy efficiency, reduced exergy destruction, and a substantial decrease in $CO_{2}$ emissions. This not only supports environmental sustainability but also aligns with global standards. Economically, the algorithm enhances the performance of the CCHP system, evident through a reduced payback period and increased annual profit. Additionally, the algorithm's rapid convergence rate allows it to reach the optimal solution faster than its counterparts, making it advantageous for time-sensitive applications. Incorporating innovative methods like chaos theory, the DNGO algorithm effectively avoids local optima, enabling a broader search for the best solution. The utilization of Lévy flight further enhances the algorithm's ability to escape local optima and navigate the search space more efficiently. Additionally, swarm intelligence is employed to simulate the collective behavior of decentralized systems, aiding in problem-solving. This research represents a significant advancement in optimization techniques for CCHP systems and offers a fresh perspective to the field of swarm-based optimization algorithms.

**Table:** Nomenclature

Symbol	Explanation	Unit
$L_{E}$	Exergy loss	–
$E_{S}$	Supplied exergy	–
$\eta_{ex}$	Efficiency of exergy	–
$Ex_{Q_{R}}$	Gas turbine heat exergy	–
$E_{x_{f}}$	The exergy of the fuel	–
$LHV_{f}$	Lowest heat value	–
$Ex_{Q_{b}}$	Generated heat exergy from the boiler,	–
$Ex_{f}^{GE}$	Exergy supplied by the gas engine	–
$Ex_{f}^{Boiler}$	Exergy supplied by the boiler	–
$Ex_{f}$	The total fuel exergy	–
$E_{L}$	The exergy leaving a system	–
$E_{S}$	The exergy entering the system	–
DE	The exergy destruction ratio	–
$H_{r}$	The necessary thermal energy	–
$E_{r}$	Electrical energy	–
$E_{G}$	The primary power network	–
$Q_{b}$	An auxiliary boiler	–
$Q_{B}$	The necessary heat	–
$Q_{R}$	The recuperated heat	–
$Q_{r}$	The quantification of heat recuperated from the gas engine	–
$F_{b}$	The fuel energy consumption	–
$\eta_{b}$	The auxiliary boiler efficiency	–
CO 2 R R	The alteration between the $CO_{2}$ emission ratios of the standard mode (NM) and the mode of CCHP scheme	–
$CDE_{NM}$	Generated $CO_{2}$ size in the normal mode	–
μ	The conversion factor emission	–
$CO_{2}^{T}$	The produced $CO_{2}$ size	–
$C_{A}$	Annual cash flow	–
$F_{\exp}$	The expenses function	–
$F_{ear}$	The earning function	–
$C_{I}$	The preliminary cost obtained by the first year	–
$B_{eu}$	The yearly equivalent uniform benefit	–
$I_{EU}$	The yearly equivalent uniform income	–
$C_{EU}$	The yearly cost of an equivalent uniform	–
i	The ratio of interest	–
n	The operational years	–
$\eta_{T}$	The thermal efficiency	–
$E_{P}$	The energy consumed by the gas turbine	–
ECF	The average national spot to source the factors of energy transformation for electricity	–
FCF	The average national spot to source the factors of energy transformation for electricity natural gas	–
$F_{GE}$	The fuel usage of a gas turbine	–
$F_{Total}$	Entire fuel energy	–
$F_{b}$	The fuel consumption of the boiler	–
$\eta_{GE}$	Gas turbine performance [1]	%
i	The Interest rate [2]	%
$\eta_{b}$	Boiler performance [3]	%
$T_{Ex,b}$	The boiler exhaust temperature [1]	°C
$T_{O}$	The oil temperature in the engine [2]	°C
$T_{Ex,GE}$	The engine exhaust temperature [2]	°C
$T_{j}$	The jacketing water temperature in the engine [2]	°C
$\mu_{CO_{2},e}$	The exchange factor of $CO_{2}$ emission for electricity [4]	$\frac{kg_{CO_{2}}}{kWh}$
$\mu_{CO_{2},NG}$	The exchange factor of $CO_{2}$ emission for natural gas [4]	$\frac{kg_{CO_{2}}}{kWh}$
$LHV_{f}$	The fuel lower heating amount [5]	$\frac{kj}{kg}$
FCF	The national mean spot to source factors of energy exchange for natural gas [6]	–
ECF	The national mean spot to source factors of energy exchange for electricity [6]	–
$F_{s}$	The price of exported electrical energy to the network [7]	$\frac{\$}{kWh}$
$F_{b}$	The price of imported electrical energy from the network [7]	$\frac{\$}{kWh}$
$F_{P}$	The price of emitted pollutant [5]	$\frac{\$}{kWh}$
T	The total value	–
$F_{x_{i}}$	The maximum value for the function	–

## 1 Introduction

CCHP systems (Combined Heating, Cooling, and Power) refer to a class of power systems that offer diverse forms of energy concurrently to a given structure. CCHP systems have gained increasing attention due to their capability for refining energy efficacy and decreasing emissions of greenhouse gas in residential buildings. This encompasses various utilities, such as electrical power, cooling, and heating. The process of optimizing CCHP system sizing in residential erections entails identifying the optimal system size that would yield maximum efficiency while minimizing energy wastage [8].

The optimization procedure encompasses the evaluation of diverse factors such as the energy demand and usage patterns of the edifice, along with the accessible resources for producing electricity and heat [9]. The objective is to determine the most favorable capacity of the CCHP system that can efficiently cater to the energy demands of the structure while reducing inefficiencies [10].

Optimization of the CCHP system sizing in housings can result in a reduction of power costs and environmental impact [11]. The aforementioned methodology has the potential to enhance resource utilization and bolster resilience in the event of power outages and other energy grid disturbances [12].

Metaheuristics represent a category of optimization algorithms that are frequently employed for resolving intricate problems such as the sizing of CCHP systems [13]. The algorithms are specifically crafted to navigate an extensive search area and identify the most favorable solution through a process of incremental enhancement of prospective solutions. Metaheuristics prove to be highly advantageous in scenarios where the problem at hand exhibits non-linearity and non-convexity; moreover, it entails the presence of multiple objectives [14].

The process of optimizing the sizing of CCHP systems in residential buildings through the use of metaheuristics entails the utilization of a metaheuristic algorithm [15].

Several works have been presented in this direction to make an improvement in the efficiency of the CCHP system. For example, Wang et al. [16] presented an approach for a CCHP system that was on the basis of solar distributed hybrid technology. The proposed system utilized panels of PV/T (Photovoltaic/Thermal) for its operation [17]. For the system, the optimal sizing was gained through a multi-objective optimization problem formulation at the upper level, which takes into account the factors of cost, energy, and environmental performance. The operational approach was determined by a mixed-integer linear programming model of lower complexity [18]. The upper-level problem was addressed through the utilization of Non-Dominated Sorting Genetic Algorithm-II. The ultimate optimum sizing was, then, determined by means of Fuzzy Decision Theory [19]. The findings indicated that the PV/T-CCHP system offers cost savings, primary energy conservation, and a reduction in carbon emissions in comparison with conventional separated production systems. The quantitative evaluation of system performance is conducted regarding the effects of solar power and equipment of energy saving (storage).

Liu et al. [20] suggested the implementation of a CCHP system for the primary structure of a gas supply enterprise located in Iran. Prior to this, the building relied on a separated generation system. The process of optimizing the ability of the prime force was undertaken with a focus on various factors, such as environmental impact, economic viability, energy efficacy, as well as exergy considerations. This was achieved through the utilization of a sophisticated optimizer, namely the amended slime mold procedure. Upon comparison with other optimization algorithms, the proposed algorithm was determined to exhibit greater accuracy and consistency. The optimal capacity of the primary driving mechanism was determined to be 78.146 kW. Sensitivity analyses were carried out on the evaluation criteria and sub-criteria. The present study offered a comprehensive methodology for the optimization of CCHP systems, which could be utilized as a valuable resource for designers of such systems.

Lucarelli et al. [21] suggested the utilization of a novel multi-objective optimizing technique for tri-generative systems of power generation, which was predicated on 3 distinct cost functions, including economic, environmental, and technical. The optimizer under consideration was employed for modeling the day-to-day functioning of multiple tri-generative system setups, resulting in a cumulative 31,680 modeling. Subsequently, a meticulous analysis of the gathered information was conducted, followed by a comparative assessment of the 22 technology sets under study. The evaluation was based on various factors, including engines that were internal ignition, absorption and compression gas heat-pumps, 2 types of fuel cells proton exchange membrane and solid oxide, as well as lead-acid and lithium-ion batteries. The present studying case pertained to a significant consumer in industry, wherein the mean load of electricity during 4 reference epochs was recorded as 4970 $\times 10^{3}$ W. The most suitable tri-generation facility for such particular consumer was determined to be composed of a 5 MWe solid oxide fuel cell powered by methane, a system of lithium-ion storing with a maximum capacity of 5 $\times 10^{5}$ W, a gas compression and an absorption heat pumps both with capacities of 39 $\times 10^{4}$ W for the production of cooling energy, and a heat pump of gas compression with 16 $\times 10^{5}$ W capacity for the production of thermal energy.

Anderson [22] introduced an approach for achieving optimal design of a CCHP system. The focal point of the present investigation was a gaseous-fueled turbine, specifically tailored for deployment in a remote region situated in Zhaoping County, China, and Guangxi. The efficacy evaluation of the procedure was predicated upon four primary parameters, namely economic, energetic, environmental, and exergetic characteristics. The Modified Group Teaching method of optimizing (MGTO) was employed to attain outcomes that exhibited superior precision and convergence. The comparative examination of the efficacy of the offered MGTO-based method was presented vis-à-vis the genetic and amended owl optimizer-based techniques. The results demonstrated the superior efficiency of the MGTO-based method.

Tooryan et al. [23] studied the optimal configuration and energy management of a hybrid microgrid system, integrating various distributed energy resources like battery, thermal power saving units, boiler, fuel cell, and photovoltaic arrays. The goal was to minimize operational costs while meeting electrical, heating, and cooling demands. The Particle Swarm Optimization Algorithm was used to determine the optimal energy management strategy and capacity allocation for each resource. The study found that utilizing residential and municipal waste reduced natural gas consumption by fuel cells and significantly reduced $CO_{2}$ emissions when heat was used by fuel cells. This validated the proposed optimal power management strategy for integrated microgrids.

Existing optimization algorithms for CCHP system sizing have limitations, such as premature convergence and local optimum issues, which can lead to suboptimal solutions. There is a need for a more effective optimization algorithm that addresses these limitations and incorporates a comprehensive 4E analysis [24]. Thus, this paper's main point is to fill the gap of research by proposing a novel methodology for optimum size of gas engine CCHP systems in residential homes in China, incorporating a comprehensive 4E analysis. The Developed Northern Goshawk Optimization (DNGO) algorithm is introduced to address the limitations of existing algorithms, providing a more cost-effective solution for CCHP system's sizing. This research contributes to the advancement of sustainable energy management and reduced environmental impact in residential buildings.

In this paper, an innovative algorithm has been introduced, named Developed Northern Goshawk Optimization (DNGO), which integrates chaos theory, Lévy flight, and swarm intelligence to optimize the gas engine's size in a CCHP system. The present study includes a thorough 4E analysis, taking into account the energy, exergy, economic, and environmental aspects of the system. The effectiveness and implications of the present approach has been evaluated at a specific site in China, and it has been compared with two other algorithms, specifically Northern Goshawk Optimization (NGO) and Genetic Algorithm (GA). The findings illustrate that the method can attain the optimal gas engine's size, resulting in the highest energy efficiency, the lowest exergy destruction ratio, and the lowest $CO_{2}$ emission reduction ratio when compared to other two algorithms. Main novelties of this work include:

- Providing a Gas Engine-CCHP for 4E optimizing design and its analysis.
- Validating the effectiveness of the proposed strategy for integrated microgrids.
- A Developed Northern Goshawk Optimizer (DNGO) for this purpose.
- Use of Energy, Exergy, Economic, and Environment (4E) analysis for performance assessment.

## 2 Methods and materials

### 2.1 The overall outline of the CCHP system

The combined cooling, heating, and power (CCHP) system is a highly efficient energy system that has the potential to enhance energy conservation and reduce carbon emissions. Through the utilization of a single primary mover, like a gas engine, to generate electricity, cooling, and heating concurrently, the CCHP system can attain superior overall efficiency and minimize environmental harm compared to traditional independent generation systems.

The present study investigates the CCHP system, which has been a form of distributed power resource that supplies electrical energy, cooling, and heating for buildings through a singular primary mover, namely a gas engine. The gas engine operates over the natural gas combustion, resulting in the generation of mechanical energy that is harnessed to power an electric generator, thereby producing electrical energy. The system is equipped with a boiler that is powered by natural gas and generates suitable steam for various purposes, including but not limited to heating.

The CCHP system utilizes the gas engine's wasted heat, encompassing the oil cooling, exhaust, and jacketing, to facilitate the provision of cooling and heating services to the housings it serves. The captured waste heat is subsequently transferred to cooling and heating equipment, thereby enabling the production of warm water or steam that could be employed for cooling or heating uses [25].

Furthermore, the CCHP system has been interconnected with the primary energy grid, enabling the transfer of energy between the system and the grid. In the event that the electrical power has been created via the CCHP system lacks the necessary load, it will proceed to obtain energy from the primary grid. In contrast, in cases where the produced power exceeds the necessary load, the surplus power can be retailed to the primary power network.

In the CCHP system, firstly, the gas engine operates by combusting natural gas to produce mechanical energy. This mechanical energy is, then, used to drive an electric generator, which generates electrical energy. Additionally, the gas engine also generates waste heat from various sources, such as oil cooling, exhaust, and jacketing. This waste heat can be effectively utilized for cooling and heating purposes.

The system also includes a boiler, which is powered by natural gas. The boiler produces steam that can be used for various heating applications, including space heating, water heating, and process heating.

Furthermore, the CCHP system incorporates heating equipment that receives the waste heat from the gas engine. This heating equipment is responsible for producing warm water or steam, which can be utilized for heating purposes. The heating equipment can take the form of a heat exchanger, a heat pump, or a steam turbine.

Similarly, the system includes cooling equipment that also utilizes the waste heat from the gas engine. This cooling equipment is responsible for producing chilled water or ice, which can be used for cooling purposes. The cooling equipment can be an absorption chiller, an adsorption chiller, or a vapor compression chiller.

**Fig. 1:**
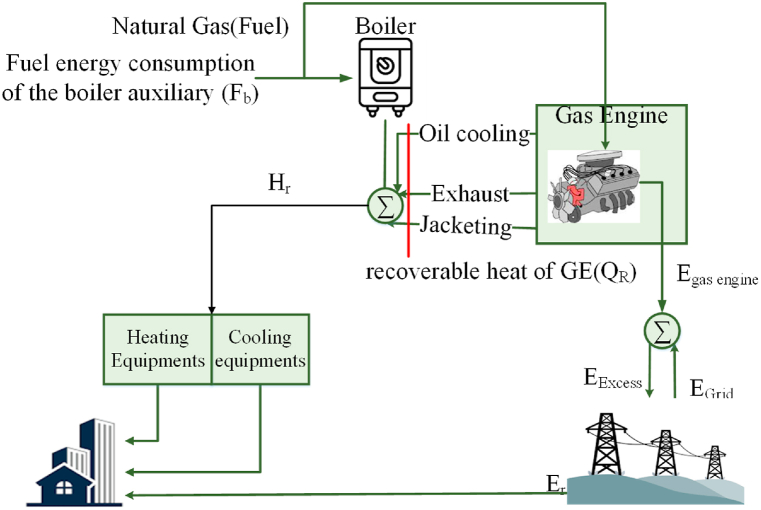
Overall outline of a gas engine-based CCHP system, as proposed by the authors.

Lastly, the CCHP system is interconnected with the primary energy grid. This interconnection allows the system to exchange energy with the grid. If the electrical power generated by the CCHP system is not sufficient to meet the load demand, it can obtain additional power from the grid. Fig. (1) displays the overall outline of the CCHP system that has been considered to be based on a gas engine, as proposed by the authors.

There are numerous advantages associated with the implementation of this CCHP system. The waste heat use for the purpose of heating and cooling results in an increased overall efficacy of the system and a reduction in the emission of greenhouse gases. The utilization of a solitary primary mover streamlines the system's design and diminishes the expenses associated with maintenance. Moreover, the capacity to transfer energy with the power grid discusses economic advantages, including diminished energy expenses and probable income from retailing surplus electricity to the grid [26].

### 2.2 Case study

The present study considers Yantian District for the CCHP system designing. The Yantian District is situated in the southeastern region of the city of Shenzhen, located in the Guangdong Province of China. The region experiences swift expansion and accommodates numerous industrial parks, among which is the Yantian Port Free Trade Zone, recognized as one of the most bustling ports globally.

The Yantian District presents a favorable location for the development of CCHP systems because of various factors. Initially, it is noteworthy that the district exhibits a substantial requirement for energy as a result of its expeditiously expanding industrial and commercial domains. Consequently, there exists a substantial requirement for effective and enduring energy alternatives that can cater to this exigency while concurrently mitigating the discharge of greenhouse gases.

Furthermore, the Yantian District exhibits continuous temperate climatic conditions, characterized by an average temperature varied from 15 °C to 30 °C. The aforementioned site presents a favorable prospect for the implementation of CCHP systems. These systems have the capability to furnish buildings with both heating and cooling functionalities by utilizing residual heat from the primary energy converter, such as a gas turbine in this context.

Moreover, the Yantian District possesses the capability to utilize natural gas, a crucial resource of energy for gas turbines that are employed in CCHP systems. The province of Guangdong has made significant investments toward the advancement of natural gas infrastructure, thereby enhancing the accessibility and cost-effectiveness of natural gas for industrial and commercial users within the region.

Yantian District is situated in close proximity to various academic and research institutions that have a specific focus on energy and sustainability, including the Shenzhen Institute of Building Research. This facilitates the acquisition of research and development of proficiency that can be utilized to devise and enhance CCHP systems tailored to the distinctive energy requisites of the locality.

**Fig. 2:**
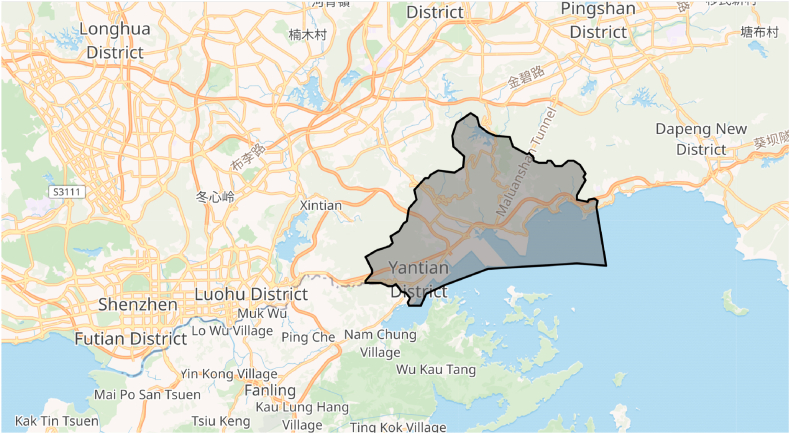
Geographical position of the Yantian District shown on Google Maps.

The structure encompasses a total floor area measuring 10,000 $m^{2}$ and comprises 200 apartments. It is equipped with a Combined Cooling, Heating, and Power (CCHP) system that utilizes a gas engine as its primary energy source. This system efficiently provides electricity, heating, and cooling to the residents. Additionally, the building is connected to the main power grid, allowing for the import or export of electricity based on the demand and supply requirements. This building serves as a representative example of the typical residential structures in China, which significantly contribute to the country's energy consumption and $CO_{2}$ emissions. Furthermore, it is situated in a region characterized by a cold and dry climate, which impacts the energy demand and supply patterns of the building. In the present analysis, the technical requirements of the region have been taken into account, including factors such as population and energy consumption. In general, the Yantian District exhibits potential as a viable site for the development of CCHP systems that could efficiently cater to the escalating energy requirements of the expanding industrial and commercial domains, while simultaneously mitigating the release of harmful greenhouse gases. The favorable climatic conditions, availability of natural gas, and close proximity to research institutions render the district to a prime site for the advancement of sustainable energy solutions. Fig. (2) displays the geographical position of the Yantian District as shown on Google Maps.

For developing the CCHP system, it has been imperative to attain comprehensive data pertaining to the load demand, encompassing both the primary structure and associated equipment. The current case study pertains to a basic structure equipped with a fan of cooling tower, boilers, and an absorption chiller. Table 1 outlines the primary variables of the machinery utilized for the simulation.

**Table 1:** Primary variables of the machinery utilized for the simulation.

Parameter	Value	Unit
Load demand of building	500	kW
Efficiency of gas turbine	35	%
Capacity of boiler	400	kW
Efficiency of boiler	85	%
Capacity of absorption chiller	300	kW
Absorption chiller COP	0.7	–
Capacity of vapor compression chiller	100	kW
Vapor compression chiller COP	3.5	–
Capacity of cooling tower fan	50	kW
Efficiency of cooling tower fan	80	%
Efficiency of overall system	70	%

### 2.3 The 4E examination of the CCHP system

In this study, the aim was to calculate the optimal sizing of a gas engine that is the prime motivator of a CCHP system in Yantian District, considering the 4E parameters, namely exergy, energy, environmental, and economic. The optimization process involves the use of metaheuristic techniques to analyze various performance indicators. A detailed explanation of the process is given below.

The initial stage of optimization involves the formulation of the problem, which necessitates the identification of the cost function and restrictions. The aim of the present study is to optimize the efficiency of the CCHP system by considering the four key performance indicators, commonly referred to as the 4E parameters. The limitations encompass various factors, such as energy and exergy efficacy, consumption of fuel, CDE (Carbon Dioxide Emissions), EDR (Exergy Destruction Rate), $CO_{2}$ RR (Reduction Rate of $CO_{2}$), PB (Payback Period), and EUAB (Equivalent Uniform Annual Benefit).

The process of system modeling involves creating a representation of a system using various techniques and tools. This representation can formulate a mathematical representation of the CCHP system, encompassing the gas engine, unit of heat recovery, absorption chiller, and supplementary apparatus. The proposed model aims to elucidate the interrelationships among the input variables, such as size of gas engine and fuel consumption, and the output variables, including efficacies of exergy and energy, environmental impact, and monetary feasibility.

Metaheuristic optimization techniques refer to a class of computational methods that are designed to solve complex optimization problems. These techniques are characterized by their ability to choose suitable metaheuristic algorithms for addressing the optimization problem. Metaheuristics have been identified as efficacious instruments for resolving intricate, non-linear, and multi-faceted optimization difficulties. The aforementioned algorithms conduct a search within the solution space with the aim of identifying the size of gas engine that is optimal, while also satisfying the constraints and objective function(s).

Implementing an algorithm involves executing the selected metaheuristic algorithms using a suitable programming language. This process includes specifying the algorithm's parameters and establishing the optimization process. The optimization process refers to the systematic approach of improving a system or process to achieve the best possible outcome, often executing the metaheuristic algorithms for exploring and identifying the most optimum result. The process entails a repetitive assessment of potential solutions, followed by the modification of the solution population, and the utilization of variation operators, such as mutation and crossover, to investigate the solution space. The optimization procedure persists until a specified termination criterion is attained, like reaching the maximum number of repetitions or meeting the convergence threshold.

**Algorithm 1:** The pseudocode of the 4E examination

1)Initialize variables for energy, exergy, economic, and environmental factors. 2)Calculate the energy generated by the system: Sum the energy produced by the GT (Gas Turbine) and the Rankine cycle. Store the result in the “energy” variable. 3)Calculate the efficiency of exergy: compute the exergy of the energy produced by the system. Divide the exergy by the energy to obtain the efficiency of exergy. Store the result in the “exergy_efficiency” variable. 4)Calculate the destruction rate of exergy: Compute the destruction rate of exergy using the methodology described in the manuscript. Store the result in the “exergy_destruction_rate” variable. 5)Analyze the energy: Compute the energy conversion efficiency of the GT and the Rankine cycle. Store the results in the “GT_efficiency” and “Rankine_cycle_efficiency” variables. Calculate the overall energy efficiency of the system by multiplying the GT and Rankine cycle efficiencies. Store the result in the “energy_efficiency” variable. 6)Environmental study: Compute the emissions of the system, such as $CO_{2}$, NOx, SOx, and particulate matter. Calculate the environmental impact of the system using a Life Cycle Assessment (LCA) methodology. Store the results in the “emissions” and “environmental_impact” variables. 7)Economic study: Compute the Capital Expenditure (CAPEX) and Operational Expenditure (OPEX) of the system. Calculate the payback period and the Equivalent Uniform Annual Benefit (EUAB) using the methods described in the manuscript. Store the results in the “CAPEX”, “OPEX”, “payback_period”, and “EUAB” variables. Print the results: 8)Print the values of energy, exergy efficiency, exergy destruction rate, energy efficiency, emissions, environmental impact, CAPEX, OPEX, Payback Period, and EUAB.

**Fig. 3:**
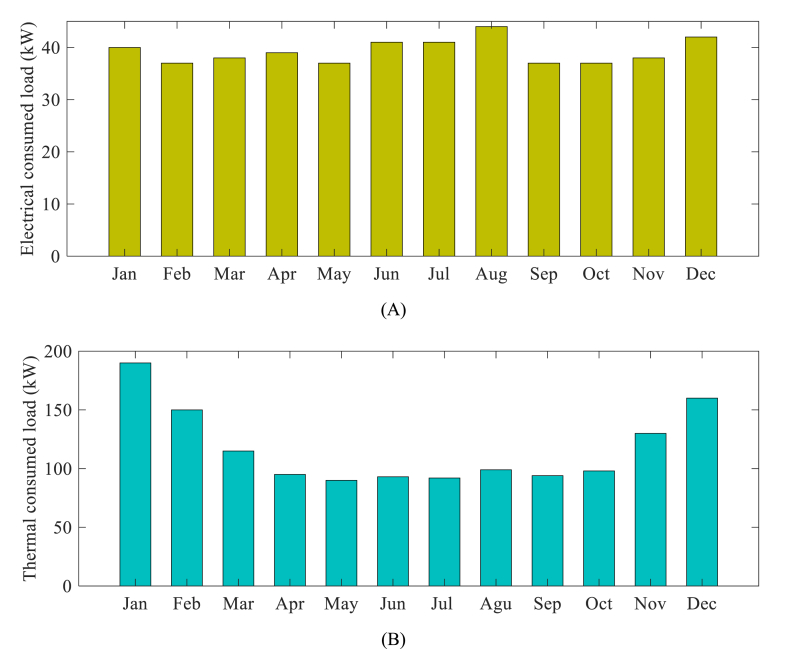
Monthly electrical and thermal loads of the residential building: (A) Electrical load (kW), (B) Thermal load (kW).

Fig. (3 (A-B)) illustrates the bar drawing for both thermal and electrical loads that have been consumed. The diagram is shown in two parts, A and B, representing electrical and thermal loads, respectively.

Fig. (3-A) depicts the monthly electrical load of the residential building measured in kilowatts (kW), whereas (3-B) illustrates the monthly thermal load of the same building, also measured in kilowatts (kW). The thermal load signifies the quantity of necessary heat energy for heating or cooling purposes within the building. Consequently, Fig. (3-B) offers insights into the fluctuations in the monthly thermal load of the building over the specified frame time. Fig. (3-B) illustrates the highest thermal load consumption occurring in January, with a peak of nearly 200 kW. Subsequently, there is a gradual decrease in the following months, reaching its lowest point in June. However, the consumption starts to rise again towards the end of the year, with December exhibiting the second-highest consumption at approximately 175 kW. This visual representation plays a vital role in comprehending the fluctuating energy demands throughout the year, which is crucial for optimizing the operation of the CCHP system and efficiently meeting the varying thermal energy requirements.

**Fig. 4:**
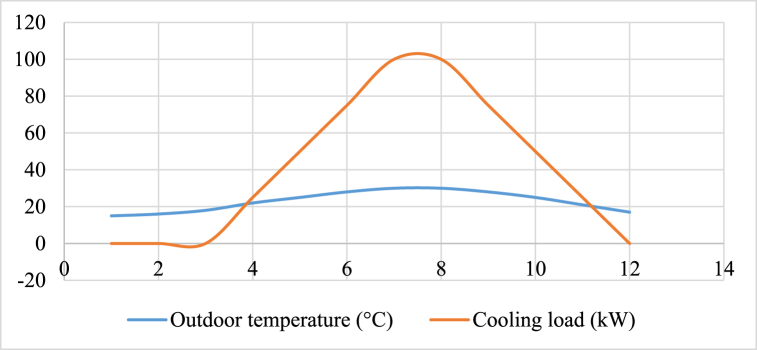
Monthly outdoor temperature and cooling load demand for the residential building in the Yantian District.

Based on the information provided in the table and utilizing standard values found in literature, the cooling load necessary for the residential building in Yantian District can be computed for every month. The results of this calculation are displayed in the table below, with a cooling setpoint of 26 °C and a ventilation rate of 0.5 [Fig (4)].

The data presented in the table indicates that the cooling load demand for the residential building in the Yantian District fluctuates between 0 and 100 kW, depending on the outdoor temperature. The highest cooling load is observed during the months of July and August, coinciding with an outdoor temperature of 30 °C. Conversely, the cooling load is non-existent in January, February, March, and December, when the outdoor temperature falls below the cooling setpoint. During the months of April, May, June, September, October, and November, the cooling load is moderate as the outdoor temperature ranges between the cooling setpoint and 30 °C.

## 3 E assessment

The study employed a methodology to analyze a system that employs a GT (Gas Turbine) as the prime means of energy generation. The study utilizes the 4E framework, consisting of four essential components, namely energy, exergy, economic, and environmental factors.

The term “energy” pertains to the aggregate quantity of energy generated by a given system, whereas “exergy” denotes the proportion of energy that can be transformed into practical work. Economic considerations encompass a range of expenses related to the setup, upkeep, and functioning of the system, in addition to the income generated by the sale of energy. Environmental factors encompass the effects of a given system on the natural surroundings, such as the emission of carbon and other harmful substances.

The investigation employs a Rankine cycle, a thermodynamic cycle commonly utilized for energy conversion, and organic fluids, which serve as the cycle working stream. The main contribution of the system focuses on concurrently producing electricity and thermal power by harnessing solar radiation.

The subsequent section of the manuscript delineates the analytical methodologies employed in the investigation to scrutinize each of the 4E constituents. The aforementioned task is expected to entail the utilization of modeling and simulations to appraise the energy and exergy efficacy of the system, and to ascertain the system's economic viability. Additionally, an assessment of the ecological impact of the system is also anticipated.

The primary aim of this investigation is to make an entire feasibility analysis of employing a gas turbine and organic fluids in a Rankine cycle for the purpose of co-generating electricity and thermal power via solar radiation. The implementation of the 4E framework guarantees that the examination incorporates the diverse elements that contribute to the efficiency and durability of the previously mentioned system.

### 3.1 Exergy efficiency

This factor is a measure of the portion of energy that can be converted into useful work, its formula is derived by taking into account the 2nd rule of thermodynamics. Equation. (1) defines the way that exergy efficiency is mathematically calculated [27].

(1) $$\eta_{ex}=1-{L_{E}/E}_{S}$$

where, $L_{E}$ refers to exergy loss, while $E_{S}$ represents supplied exergy.

When analyzing Fig. (1) in line with the offered CCHP system, the exergy efficiency is given by Equation. (2):

(2) $$\eta_{ex}=\frac{E_{r}+E_{E}+Ex_{Q_{R}}+Ex_{Q_{b}}}{E_{G}}\times 100$$

The formula above is based on various energy flows. The gas turbine heat exergy that is Recoverable has been represented by $Ex_{Q_{R}}$ and is calculated using Equation. (3) [28]:

(3) $$Ex_{Q_{R}}=Q_{EH}\times \left(1-\frac{T_{0}}{T_{EX/GE}}\right)+Q_{j}\times \left(1-\frac{T_{0}}{T_{j}}\right)+Q_{O}\times \left(1-\frac{T_{0}}{T_{O}}\right)$$

where, $Q_{EH}$ describes the heat transferred from the exhaust gas of the gas engine to the evaporator of the absorption chiller, $T_{0}$ defines the ambient temperature, $T_{EX/GE}$ specifies the temperature of the exhaust gas of the gas engine, $Q_{j}$ determines the heat transferred from the jacket water of the gas engine to the heating equipment, $T_{j}$ represents the temperature of the jacket water of the gas engine, $Q_{O}$ signifies the heat transferred from the oil cooling system of the gas engine to the heating equipment, $T_{O}$ determines the temperature of the oil cooling system of the gas engine.

It includes three separate terms that correspond to different sources of exergy loss. The exergy of the fuel, $E_{x_{f}}$, is expressed in Equation. (4) as the production of fuel mass flow ratio and lowest heat value [29]:

(4) $$Ex_{f}=LHV_{f}\times {\dot{m}}_{f}$$

where, $LHV_{f}$ describes the lower heating value of the fuel, measured in kJ/kg, and ${\dot{m}}_{f}$ determines the mass flow rate of the fuel.

Generated heat exergy from the boiler, $Ex_{Q_{b}}$, which is obtained through Equation. (5), also contributes to the total exergy efficiency [30].

(5) $$Ex_{Q_{b}}=\frac{\left(T_{Ex/b}-T_{0}\right)\times Q_{b}}{T_{Ex/b}}$$

where, $T_{Ex/b}$ determines the temperature of the exhaust gas from the boiler, and $Q_{b}$ defines the heat transfer rate from the boiler.

Equation. (6) reveals that the total fuel exergy is equivalent to the sum of exergy supplied by the gas engine and by the boiler [31].

(6) $$Ex_{f}=Ex_{f}^{GE}+Ex_{f}^{Boiler}$$

By using these equations, it is possible to compute the efficiency of exergy for the CCHP system, which provides insights into the system's performance and sustainability.

### 3.2 Destruction rate of exergy

This section elucidates the exergy destruction ratio, a metric employed to identify the components of a system where exergy is being dissipated, as a gauge of resource degradation. Exergy is a metric that quantifies the energy's quality and denotes the highest quantity of beneficial work that can be derived from a specific quantity of energy. The phenomenon of exergy destruction is observed when there is a conversion of high-grade energy into low-grade energy, which can be exemplified by the transfer of heat from a hotter object to a relatively cooler one. equation (7) is utilized to compute the exergy destruction ratio [32].

(7) $$DE=E_{L}\times E_{S}^{-1}$$

where, $E_{L}$ and $E_{S}$ represent the exergy leaving a system and entering the system, respectively.

### 3.3 Analyzing the energy

The present concept is founded on the fundamental principles of thermodynamics and entails an analysis of the power usage and producing the diverse constituents of a CCHP framework. The illustration depicted in Fig. (1) demonstrates that the gas engine provides the necessary thermal energy ($H_{r}$) and electrical energy ($E_{r}$) to the network, and any surplus energy is transmitted to the primary power network ($E_{G}$). Conversely, in the event that the gas engine fails in generation of sufficient electrical power to fulfill the requisite demand, the electricity is procured from the grid. The correlation between the electrical power supplied via the gas engine and Equation. (8) is demonstrated in the following formula [33].

(8) $$E_{G}=\left\{ \begin{array}{c} E_{r}-E_{GE},{E_{GE}<E}_{r}\\ E_{GE}-E_{r},{E_{GE}>E}_{r} \end{array}\right.$$

In the event that supplementary thermal energy is required, it is possible to utilize an auxiliary boiler ($Q_{b}$) to produce it [33] [Equation. (9)].

(9) $$Q_{b}=Q_{B}-Q_{R},Q_{R}<Q_{B}$$

According to Eq. (9), $Q_{b}$ can be expressed as the disparity between the necessary heat ($Q_{B}$) and the recuperated heat ($Q_{R}$).

The quantification of heat recuperated from the gas engine is determined through the utilization of Equation. (10), which takes into account the exhaust heat output, produced via the oil chilling system, and produced by the jacketing system, with corresponding coefficients of 0.5, 0.35, and 0.15.

(10) $$Q_{r}=0.5\times Q_{EH}+0.35\times Q_{j}+{0.15\times Q}_{O}$$

The assessment of boiler performance is conducted through the utilization of Equation (11).

(11) $$\eta_{b}=Q_{b}/F_{b}$$

where, it takes into account the auxiliary boiler efficiency denoted by $\eta_{b}$ and the fuel energy consumption $F_{b}$ as determined by Equation. (12) that incorporates the lower heating value of the fuel.

(12) $$F_{b}={\dot{m}}_{b}\times LHV_{f}$$

where, ${\dot{m}}_{b}$ describes the mass flow rate of the fuel of the boiler, and $LHV_{f}$ signifies the lower heating value of the fuel.

As elucidated in Ref. [34], the primary limitations of energy examination pertain to the overall efficacy of energy as well as fuel energy usage.

### 3.4 Environmental study

The production of $CO_{2}$ has been a major contributor to emissions of greenhouse gas, and it results primarily from the natural gas combustion in the gas engine and boiler. Therefore, minimizing the amount of $CO_{2}$ generated is a key factor in selecting a CCHP system.

The $CO_{2}$ RR element refers to the alteration between the $CO_{2}$ emission ratios of the standard mode (NM) and the mode of CCHP scheme. In the standard mode (NM), electricity from the main grid meets the load demand using an equal amount of natural gas. The $CO_{2}$ RR is calculated using equation (13):

(13) $$CO_{2}RR=\left(CDE_{NM}-CDE\right)/CDE_{NM}$$

where, CDE describes the $CO_{2}$ emission of the CCHP system, and $CDE_{NM}$ specifies size of generated $CO_{2}$ in the normal mode attained via the formula below [Equation. (14)]:

(14) $$CDE_{NM}=\mu_{CO_{2},E}\times E_{B}+\mu_{CO_{2},NG}\times F_{B}$$

where, μ specifies the conversion factor emission. During this, the size of the produced $CO_{2}$ can be mathematically achieved as follows [Equation. (15)]:

(15) $$CO_{2}^{T}=CO_{2,gas\;engine}+CO_{2,Boiler}+CO_{2,grid}-CO_{2,excess}$$

Moreover, the capacity of $Em_{CO_{2}}$ provides a different operative constraint which has been achieved as follows [Equation. (16)]:

(16) $$Em_{CO_{2}}=\mu_{CO_{2},f}{\times F}_{GE}+{\mu_{CO_{2},f_{b}}{\times F}_{b}+\mu }_{CO_{2},E}{\times (E}_{G}-E_{E})$$

where, $\mu_{CO_{2},f}$ signifies the $CO_{2}$ emission factor of the fuel, $F_{GE}$ determines the fuel consumption of the gas engine, $F_{b}$ specifies the fuel consumption of the boiler, $\mu_{CO_{2},E}$ signifies the $CO_{2}$ emission factor of the electricity, $E_{G}$ describes the electricity generation of the CCHP system, and $E_{E}$ describes the electricity consumption of the CCHP system, and $\mu_{CO_{2},f_{b}}$ represents the $CO_{2}$ emission factor of the fuel for the boiler that is achieved as follows [Equation. (17)]:

(17) $$\mu_{CO_{2},f_{b}}=\mu_{CO_{2},f_{GE}}=\mu_{CO_{2},NG}$$

### 3.5 Economic study payback period

The cash stream of the system refers to an analysis of its performance, which involves calculating the annual cash flow ($C_{A}$) to determine the system net profit throughout its lifespan. It is achieved by subtracting the expenses function ($F_{\exp}$) from the earning function ($F_{ear}$), as expressed in Ref. [35] [Equations. (18-20)]:

(18) $$C_{A}=\sum_{i=1}^{y}\sum_{y=1}^{n}\left[F_{ear}-F_{\exp}\right],y=1,2,\ldots ,n..$$

Such that:

(19) $$F_{ear}=F_{E}+F_{env}+{\left(F_{H}\right)}_{GE}$$

(20) $$F_{\exp}=C_{I}+F_{OM}{+\left(F_{F}\right)}_{Boiler}+F_{G}+{\left(F_{F}\right)}_{GE}$$

where, $C_{I}$ specifies the preliminary cost obtained by the first year.

### 3.6 Equivalent uniform annual benefit

The yearly equivalent uniform benefit ($B_{eu}$) is a measure of the actual monetary value of a system, determined by computing its annual costs and incomes throughout the time, taking into account the interest rate. $B_{eu}$ is given by equation (21):

(21) $$B_{eu}=I_{EU}-C_{EU}$$

where, $I_{EU}$ and $C_{EU}$ represent the yearly equivalent uniform income, and the yearly cost of equivalent uniform as calculated in equations (22), (23) [2]:

(22) $$I_{EU}=F_{E}+F_{env}+F_{H}^{GE}+\frac{C_{I}\times i}{{\left(1+i\right)}^{n}-1}$$

(23) $$C_{EU}=\frac{C_{I}\times i\times {\left(1+i\right)}^{n}}{{\left(1+i\right)}^{n}-1}+F_{G}{+F}_{OM}+F_{F}^{Boiler}+F_{F}^{GE}$$

where, i stands for the interest rate, and n defines the lifetime of the CCHP system. $F_{E}$ describes the fuel cost of the CCHP system, $F_{env}$ specifies the environmental cost of the CCHP system, $F_{H}^{GE}$ represents the maintenance cost of the gas engine, $C_{I}$ describes the initial investment cost of the CCHP system, and incorporating costs related to $F_{G}$, $F_{OM}$, $F_{F}^{Boiler}$, and $F_{F}^{GE}$ in addition to the ratio of interest (i) and the operational years (n).

The economic justification of the system relies on the calculation of the payback period. The term refers to the duration that is necessary to offset the initial annual capital revenues. The present term can be derived through the utilization of equation (24) [7]:

(24) $$P_{p}=C_{I}/cf$$

The overall efficiency pertains to energy.

Given the scheme of the examined system of CCHP, the entire energy efficacy can be attained through the utilization of the equation presented in Ref. [6].

Equation. (25) represents the calculation of the thermal efficiency ($\eta_{T}$) in a thermodynamic system, where it is determined as follows:

(25) $$\eta_{T}=\left(E_{r}+E_{E}+Q_{B}+Q_{E}\right)/E_{P}$$

The variable $E_{P}$ describes the energy consumed by the gas turbine and could be computed based on the following formula [Equation (26)]:

(26) $$E_{P}=\left(E_{G}\times ECF\right)+\left(\left(F_{b}+F_{GE}\right)\times FCF\right)$$

The variables ECF and FCF denote the average national spot to source the factors of energy transformation of electricity and natural gas. Additionally, $F_{GE}$ represents the fuel usage of a gas turbine, which can be calculated using equation (27):

(27) $$F_{GE}=\left({Q_{R}+E}_{GE}\right)/\eta_{GE}$$

Moreover, entire fuel energy usage represents the secondary scenario for evaluating the magnitude of fuel energy consumption through energy analysis. This factor encompasses the fuel consumption of both the GT and boiler, delineated as [Equation (28)]:

(28) $$F_{Total}=F_{GE}+F_{b}$$

Table 2 displays the necessary input parameters for conducting an optimal analysis of the 4E concept.

**Table 2:** Necessary input parameters for conducting an optimal analysis of the 4E concept.

Notation	Parameter	Value	Unit
$\eta_{GE}$	Gas turbine performance [1]	86	%
i	The Interest rate [2]	19	%
$\eta_{b}$	Boiler performance [3]	82	%
$T_{Ex,b}$	The boiler exhaust temperature [1]	278	°C
$T_{O}$	The oil temperature in the engine [2]	62	°C
$T_{Ex,GE}$	The engine exhaust temperature [2]	563	°C
$T_{j}$	The jacketing water temperature in the engine [2]	102	°C
$\mu_{CO_{2},e}$	The exchange factor of $CO_{2}$ emission for electricity [4]	0.92	$\frac{kg_{CO_{2}}}{kWh}$
$\mu_{CO_{2},NG}$	The exchange factor of $CO_{2}$ emission for natural gas [4]	0.23	$\frac{kg_{CO_{2}}}{kWh}$
$LHV_{f}$	The fuel lower heating amount [5]	67,670	$\frac{kj}{kg}$
FCF	The national mean spot to source factors of energy exchange for natural gas [6]	1.28	–
ECF	The national mean spot to source factors of energy exchange for electricity [6]	3.56	–
$F_{s}$	The price of exported electrical power to the network [7]	0.07	$\frac{\$}{kWh}$
$F_{b}$	The price of imported electrical energy from the network [7]	0.16	$\frac{\$}{kWh}$
$F_{P}$	The price of emitted pollutant [5]	0.037	$\frac{\$}{kWh}$

## 4 Developed Northern Goshawk Optimizing method

The offered Northern Goshawk Optimizer (NGO) has been explained in this part. Afterwards, its mathematical formulation has been illustrated.

A. Motivation and manner of Northern Goshawk

This creature is a hunter in the carnivorous birds’ family with medium-large size. This kind of bird, as a member of Accipiter, can hunt numerous kinds of prey, including large/small-sized birds, mammals with small sizes like rabbits, mice, squirrels, raccoons, and foxes [36]. The habitat of Northern goshawk has been North America and Eurasia. The female northern goshawk is slightly smaller compared to the males which has been measured 58–69 cm body length, 46–61 cm space among 2 wings, and 1.22 kg weight. The male, however, is weighted 0.78 kg, 89–105 cm length, and 108–127 cm space amid 2 wings. The attitude of Northern goshawk in hunting is a smart process and its hunting policy has 2 steps. In the 1st phase, the prey is identified by the bird who chases the target with high velocity [37]. In the 2nd phase, the prey is hunted in a process of short tail-pursuit. Mathematical simulation of the declared policy is the main motivation in design of the offered NGO.

B. Initialization process of the optimizer

The offered NGO is an algorithm based on the population in which the search individuals are northern goshawks. In this optimizer, every goshawk implies an offered result to the optimization problem that evaluates the variables’ amount. Every individual is defined as a vector which forms a matrix altogether. At the outset of the execution of the optimizer, individuals have been initialized on a random basis over the solution space. The matrix of individuals for the offered NGO has been illustrated in formula below [Equation (29)] [36].

(29) $$Y=\left[\begin{array}{c} Y_{1}\\ \vdots \\ \begin{array}{c} Y_{i}\\ \begin{array}{c} \vdots \\ Y_{N} \end{array} \end{array} \end{array}\right]=\left[\begin{array}{cc} \begin{array}{ccc} y_{1,1} & \ldots & y_{1,j} \end{array} & \begin{array}{cc} \ldots & y_{1,m} \end{array}\\ \begin{array}{ccc} \begin{array}{c} \vdots \\ y_{i,1}\\ \begin{array}{c} \vdots \\ y_{N,1} \end{array} \end{array} & \begin{array}{c} \ddots \\ \ldots \\ \begin{array}{c} \ddots \\ \ldots \end{array} \end{array} & \begin{array}{c} \vdots \\ y_{i,j}\\ \begin{array}{c} \vdots \\ y_{N,j} \end{array} \end{array} \end{array} & \begin{array}{cc} \begin{array}{c} \ddots \\ \ldots \\ \begin{array}{c} \ddots \\ \ldots \end{array} \end{array} & \begin{array}{c} \vdots \\ y_{i,m}\\ \begin{array}{c} \vdots \\ y_{N,m} \end{array} \end{array} \end{array} \end{array}\right]$$

here, Y represents the population individuals of the optimizer, $Y_{i}$ demonstrates the offered result i, $y_{i,j}$ signifies the variable j amount indicated via the offered result i. The variables' number of the problems and the individuals' number are represented by m and N, respectively.

For the problem of optimization, the cost function could be determined on the basis of every individual in population on the grounds that every individual provides a result for problem. A vector which is illustrated in the formula below can be utilized for obtaining the cost function values [Equation (30)].

(30) $$\vec{C}=\left[\begin{array}{c} C_{1}\\ \vdots \\ \begin{array}{c} C_{i}\\ \begin{array}{c} \vdots \\ C_{N} \end{array} \end{array} \end{array}\right]=\left[\begin{array}{c} {C(Y}_{1})\\ \vdots \\ \begin{array}{c} {C(Y}_{i})\\ \begin{array}{c} \vdots \\ {C(Y}_{N}) \end{array} \end{array} \end{array}\right]$$

In which, C represents a vector that includes the cost function amounts, $C_{i}$ signifies the amount of cost function gained via the offered result i.

The amount of cost function has been the main criteria in indicating the finest result of the optimization issue. In minimizing problems, in which minimizing is the aim, the smaller cost function value, the finer the offered result. For the maximizing problems, however, the larger cost function amount, the finer the offered result. As new amounts have been gained for the cost function over each epoch, the finest result ought to be renewed in every epoch.

C. Mathematical Simulation of offered NGO

In the offered NGO design, the hunting policy of northern goshawk has been modeled for renewing the population individuals. As mentioned, the hunting strategy contains 2 key manner of northern goshawk: 1) identification of the target and attack, 2) pursue and escaping act for hunting.

1) The 1st phase (Exploration): Identification of Prey

Over this phase, the Northern goshawk selects a prey on a random basis and promptly attacks that target. Due to the randomly choosing process of the target, the offered NGO exploration power gets enhanced by this step. By recognizing the optimum region, this phase causes a global search of the solution space. Fig. 1 represents the scheme of the goshawk attitude over the 1st step of hunting. The expressed concepts have been simulated based on the formulas below [Equations (31), (32), (33)]:

(31) $${\mathrm{Pr}}_{i}=Y_{k},i=\left[1,N\right],k=\left[1,\ldots ,i-1,i+1,\ldots ,N\right]$$

(32) $$y_{i,j}^{new,{\mathrm{Pr}}_{1}}=\left\{ \begin{array}{c} y_{i,j}+r\left({\mathrm{Pr}}_{i,j}-Iy_{i,j}\right),C_{{\mathrm{Pr}}_{i}}<C_{i}\\ y_{i,j}+r\left({\;y_{i,j}-\mathrm{Pr}}_{i,j}\right),Otherwise \end{array}\right.$$

(33) $$Y_{i}=\left\{ \begin{array}{c} Y_{i}^{new,{\mathrm{Pr}}_{1}},C_{i}^{new,{\mathrm{Pr}}_{1}}<C_{i}\\ Y_{i},Otherwise \end{array}\right.$$

here, the situation of the prey regarding the goshawk i has been represented by ${\mathrm{Pr}}_{i}$, and the amount of the cost function is signified by $C_{{\mathrm{Pr}}_{i}}$. k signifies a random amount varying from one to N. $Y_{i}^{new,{\mathrm{Pr}}_{1}}$ depicts the new situation of the result i, $y_{i,j}^{new,{\mathrm{Pr}}_{1}}$ defines the dimension j of the result i’s new situation, $C_{i}^{new,{\mathrm{Pr}}_{1}}$ represents its cost function amount on the basis of the 1st phase of the optimizer, r and I demonstrate random amounts, r varies from zero to one, and I can vary from one to two. These two random values have been employed for generation of random NGO attitude in searching and renewing process.

2) The 2nd Phase (Exploitation): Pursuing and escaping

Naturally, the prey attempts to escape when the goshawk attacks it. Thus, in a process of tail and pursuit, the goshawk keeps chasing the prey. Because of the goshawks’ high velocity, they are capable of pursuing their target in approximately all states and hunting it. Modeling of such an attitude causes an increase in the optimizer exploitation ability when it comes to the local search over the solution space. In the offered optimizer, this hunting has been considered adjacent to the situation of attack with R radius. Fig. 2 depicts the process of pursuing amid the prey and the goshawk. These explanations can be modeled as the following formulas [Equations (34), (35), (36)]:

(34) $$R=\frac{2}{100}\left(1-t_{m}\right)$$

(35) $$y_{i,j}^{new,{\mathrm{Pr}}_{2}}=y_{i,j}+R\left(2r-1\right)\;y_{i,j}$$

(36) $$Y_{i}=\left\{ \begin{array}{c} Y_{i}^{new,{\mathrm{Pr}}_{2}},C_{i}^{new,{\mathrm{Pr}}_{2}}<C_{i}\\ Y_{i},Otherwise \end{array}\right.$$

here, the counter of iteration is illustrated by t, and the highest number of iterations has been demonstrated by $t_{m}$. The new situation of the gained i result is represented by $Y_{i}^{new,{\mathrm{Pr}}_{2}}$, the dimension j’s new location for the result i is illustrated by $y_{i,j}^{new,{\mathrm{Pr}}_{2}}$, and $C_{i}^{new,{\mathrm{Pr}}_{2}}$ signifies the amount of cost function on the basis of the 2nd phase of the optimizer.

3) Process of Repetition for the offered NGO

One repetition of the NGO has been completed when all individuals are renewed using the 1st and 2nd phases of the offered algorithm. Afterwards, the new amounts for the individuals, cost function, and the finest result have been produced. Subsequently, NGO starts the perspective iteration, and the renewing process keeps going on till the final iteration. Eventually, the finest result gained at the end of NGO is announced as a quasi-optimum result for the optimization issue.

D. Developed Northern Goshawk Optimizing (DNGO) algorithm

The NGO algorithm mimics the goshawks’ strategies of searching, attacking, and capturing the prey; moreover, the animal uses a random parameter I to control the local and global search of the solution space. However, the NGO algorithm may suffer from premature convergence due to the fixed value of I in each iteration.

To overcome this drawback, an enhanced version of the NGO algorithm has been proposed, called the Developed Northern Goshawk Optimizing (DNGO) method, which incorporates two mechanisms to improve the performance of the NGO algorithm: the circle mapping and the Lévy flight. The circle mapping is a new chaotic map that can generate chaotic sequences with high complexity and randomness. The Lévy flight is a random walk model that can simulate the long-range jumps and the heavy-tailed distribution of some natural phenomena. The circle mapping is used to update the parameter I dynamically in each iteration, while the Lévy flight is used to adjust the step size of the goshawks’ movement. The DNGO method can enhance the diversity and convergence of the NGO algorithm and avoid being trapped in local optima.

The main steps of the DNGO method are as follows:

Initializing the population of goshawks and their positions randomly within the feasible region of the problem.

Evaluating the fitness of each goshawk using the objective function of the problem.

Updating the best goshawk (the leader) and the worst goshawk (the prey) based on their fitness values.

Updating the parameter I using the circle mapping equation.

Updating the positions of the goshawks using the Lévy flight equation.

Checking the feasibility of the new positions and repairing them if necessary.

Repeating steps 2–6 until the termination criterion is met.

The DNGO method is a general optimization technique that can be applied to various types of problems, such as continuous, discrete, or mixed-integer problems. In this paper, the DNGO method has been applied to the optimal design of a hybrid photovoltaic and fuel cell power generation system, which is a complex and nonlinear problem with multiple objectives and constraints. The DNGO method has been compared with other optimization techniques, such as Genetic Algorithm (GA), Particle Swarm Optimization (PSO), and Differential Evolution (DE), and it has been shown that the DNGO method can achieve better results in terms of solution quality, diversity, and robustness.

In the last couple of decades, several chaotic maps are being unearthed by scholars across various fields of human endeavor. Numerous mechanisms are present in various algorithms to address diverse real-world issues such as optimization.

As previously stated, the NGO algorithm incorporates a random parameter I that, when utilized in each iteration, can result in premature convergence of the algorithm. This investigation employs a chaos mechanism, specifically circle mapping, to address the aforementioned matter. The circle mechanism is utilized to transform the unidentified parameter r into a standardized formulation [Equation (37)] [38].

(37) $$I_{i+1}=I_{i}+\rho -\left(\alpha -2\pi \right)\sin\left(2\pi I_{i}\right)\;\mathrm{mod}\left(1\right)$$

here, a chaotic time sequences $r_{i}\in \left[0,1\right]$ have been produced using $\alpha =\frac{1}{2}$ and $\rho =\frac{1}{5}$.

The Lévy Flight (LF) method was employed in the present investigation to enhance convergence. The LV mechanism finds extensive application in both artificial optimization algorithms [39]. The random walk for controlling the local exploration is modeled via LV in the subsequent manner [Equations (38), (39), (40)]:

(38) $$\text{Le}\left(w\right)\approx \frac{1}{w^{1+\tau }}$$

(39) $$w=\frac{A}{{\left|B\right|}^{1/\tau }}$$

(40) $$\sigma^{2}={\left\{\frac{\Gamma \left(1+\tau \right)}{\tau \Gamma \left(\left(1+\tau \right)/2\right)}\frac{\sin\;\left(\pi \tau /2\right)}{2^{\left(1+\tau \right)/2}}\right\}}^{\frac{2}{\tau }}$$

The function Γ(.) is responsible for determining the Gamma function, while the LV mechanism index is denoted by 0≤τ≤2 (in this case, τ=3/2 [40]). The variables A and B follow a standard distribution with variance of $\sigma^{2}$ and mean of zero. The step size is represented by w. The notation $A/B∼N\left(0,\sigma^{2}\right)$ indicates that the samples are gained from a Gaussian distribution with a $\sigma^{2}$ variance and mean of zero.

The new positions of the Northern Goshawk can be derived by utilizing the LV mechanism, which can be mathematically represented by equation (41):

(41) $$y_{i,j}^{new,{\mathrm{Pr}}_{1}}=\left\{ \begin{array}{c} y_{i,j}+\text{Le}\left(\delta \right)\times \left({\mathrm{Pr}}_{i,j}-Iy_{i,j}\right),C_{{\mathrm{Pr}}_{i}}<C_{i}\\ y_{i,j}+\text{Le}\left(\delta \right)\times \left({\;y_{i,j}-\mathrm{Pr}}_{i,j}\right),Otherwise \end{array}\right.$$

E. Algorithm validation

To authenticate the effectiveness of the modified Northern Goshawk Optimization algorithm, several experiments have been conducted on standard benchmark sets. The computer configuration used for the experiments is as follows: A PC with an Intel Core i7-5600U processor of 2.6 GHZ 8.0 GB.

The results of the developed NGO have been compared with 5 recently-published metaheuristic optimizers, including Pelican Optimizing Algorithm (POA) [41], Sine Cosine Algorithm (SCA) [42], Tunicate Swarm Algorithm (TSA) [43], Whale Optimizing Approach (WOA) [44], and Salp Swarm Algorithm (SSA) [45].

Each algorithm was run 20 times on all test functions to provide reliable results. Table 3 presents data pertaining to the benchmark functions utilized for the purpose of validating the algorithms. It is worth noting that the lowest amount of all benchmarks sets is zero.

**Table 3:** Data pertaining to the benchmarks utilized for the purpose of validating the algorithms.

Test Function	formulation	Dimension	Range
Sphere	$F_{1}\left(x\right)=\sum_{i=1}^{d}x_{i}^{2}$	30	[−100, 100]
Schwefel 1.2	$F_{2}\left(x\right)=\sum_{i=1}^{d}{\left(\sum_{j=1}^{i}x_{j}^{2}\right)}^{2}$	30	[−100, 100]
Schwefel 2.20	$F_{3}\left(x\right)=\sum_{i=1}^{d}{|x}_{i}|$	30	[−100, 100]
Schwefel 2.21	$F_{4}\left(x\right)=\max_{i=1,2,\ldots ,d}{|x}_{i}|$	30	[−100, 100]
Schwefel 2.22	$F_{5}\left(x\right)=\sum_{i=1}^{n}{|X}_{i}|+\prod_{i=1}^{n}\left|x_{i}\right|$	30	[−10, 10]
Schwefel 2.23	$F_{6}\left(x\right)=\sum_{i=1}^{d}x_{i}^{10}$	30	[−10, 10]
Rosenbrock	$F_{8}\left(x\right)=\sum_{i=1}^{n-1}\left[{\left(x_{i}-1\right)}^{2}+10^{2}{\left(x_{i+1}-x_{i}^{2}\right)}^{2}\right]$	30	[-30,30]
Step	$F_{7}\left(x\right)=\sum_{i=1}^{d}{\left(x_{i}+0.5\right)}^{2}$	30	[−100, 100]
Sum Squares	$F_{9}\left(x\right)=\sum_{i=1}^{d}ix_{i}^{2}$	30	[−10, 10]
Zakharov	$F_{10}\left(x\right)=\sum_{i=1}^{d}x_{i}^{2}+\sum_{i=1}^{d}{\left(0.5ix_{i}\right)}^{2}+\sum_{i=1}^{d}{\left(0.5ix_{i}\right)}^{4}$	30	[-5,10]
Quartic	$F_{11}\left(x\right)=\sum_{i=1}^{d}x_{i}^{4}+rand\left(0,1\right)$	30	[−1.28, 1.28]
Powell sum	$F_{12}\left(x\right)=\sum_{i=1}^{d}{|x}_{i}{|}^{i+1}$	30	[−1, 1]

The benchmarks serve as widely recognized test functions utilized to assess the efficiency of optimization algorithms across various problem types. These functions exhibit diverse characteristics, including convexity, modality, separability, and scalability. They are detailed in Table 3, featuring their formulations, dimensions, and ranges. To ensure equitable comparisons, all test functions are standardized with the same dimension and range. Each algorithm undergoes 100 iterations on each test function, with data recorded for the best objective value, average objective value, standard deviation, and CPU time. Furthermore, statistical tests are conducted to evaluate the significance of algorithmic differences. The subsequent section presents the results and analysis.

The performance metrics used for comparison include the finest, poorest, standard deviation (StD), and mean of the cost function amounts obtained by each algorithm. Additionally, statistical tests have been performed to determine the significance of the differences in performance between the modified NGO and the mentioned optimizers. Table 4 indicates the set parameter amounts of each comparative algorithm.

**Table 4:** Set parameter values of each comparative algorithm.

Algorithm	Parameters	Standard Values	Ref.
POA	Pop. size, max iterations, I, R, T	30, 100, 1, 0.5, 200	[41]
SCA	Pop. size, max iterations, Search agents, $P_{\min}$, $P_{\max}$	30, 100, 80, 1, 4	[42]
TSA	Pop. size, max iterations, Search agents Number of elites	30, 100, 80, 2	[43]
WOA	Pop. size, max iterations, a, $a_{step}$	30, 100, 2, 1	[44]
SSA	Pop. size, max iterations, $c_{1}$, $c_{2}$	30, 100, 2, 2	[45]

This comparison helps to exhibit the effectiveness and competitiveness of the modified optimizer in comparison with other well-established optimization techniques. To ensure reliable results, all optimizers get run for 15 times over all test functions. The performance metrics used for comparison included the Best, Min, and StD of the cost amounts obtained by each algorithm. The outcomes were compared with five aforementioned optimizers that has been shown in Table 5.

**Table 5:** Comparison results between enhanced political optimizer and different published optimizers.

Function	indicator	POA	SCA	TSA	WOA	SSA	DNGO
F1	Best	0.00	0.00	142.51	0.00	4.58	0.00
	Min	0.00	0.00	278.35	0.00	13.57	0.00
	StD	0.00	0.00	181.05	0.00	13.49	0.00
F2	Best	45314.89	10.23	891.90	0.17	267.03	0.00
	Min	52733.17	74.90	2656.98	0.82	1012.55	0.00
	StD	18852.41	31.49	2650.59	0.91	636.59	0.00
F3	Best	0.00	0.02	44.93	0.00	4.53	0.00
	Min	0.00	0.06	67.64	0.00	18.96	0.00
	StD	0.00	0.01	14.67	0.00	9.76	0.00
F4	Best	0.03	0.24	6.11	0.00	9.64	0.00
	Min	36.28	0.27	10.37	0.00	17.61	0.00
	StD	13.26	0.12	2.94	0.00	2.49	0.00
F5	Best	0.00	0.00	6.95	0.00	0.24	0.00
	Min	0.00	0.01	6.97	0.00	2.04	0.00
	StD	0.00	0.00	1.51	0.00	1.72	0.00
F6	Best	0.00	0.00	0.18	0.00	1.46	0.00
	Min	0.00	0.00	104.15	0.00	1.32	0.00
	StD	0.00	0.00	176.01	0.00	3.58	0.00
F7	Best	0.25	1.20	134.45	0.03	1.46	0.00
	Min	1.12	1.56	400.66	0.11	13.55	0.00
	StD	0.32	0.23	121.95	0.04	14.69	0.00
F8	Best	19.37	9.98	3131.45	15.51	92.56	11.70
	Min	22.40	25.51	38458.59	19.22	1124.06	12.14
	StD	0.04	7.66	36476.53	0.30	1583.28	0.26
F9	Best	0.00	0.00	16.07	0.00	0.15	0.00
	Min	0.00	0.00	28.61	0.00	2.74	0.00
	StD	0.00	0.00	27.09	0.00	2.62	0.00
F10	Best	154.88	0.19	76.26	5.16	41.77	0.00
	Min	430.78	6.17	257.56	20.35	183.17	0.00
	StD	61.10	6.12	59.61	10.26	41.06	0.00
F11	Best	0.00	0.00	0.10	0.00	0.27	0.00
	Min	0.02	0.01	0.19	0.00	0.36	0.00
	StD	0.01	0.00	0.10	0.00	0.18	0.00
F12	Best	0.00	0.00	0.00	0.00	0.00	0.00
	Min	0.00	0.00	0.00	0.00	0.00	0.00
	StD	0.00	0.00	0.00	0.00	0.00	0.00

The results of the experiments demonstrated that the modified Northern Goshawk Optimization algorithm, incorporating the Chaotic Circle Map and Lévy Flight techniques, performed far better than the other optimizers when it came to convergence speed, result quality, and ability to escape the local optima. It validated the effectiveness of the proposed modifications in enhancing the performance of the NGO algorithm for tackling complicated problems of optimization.

## 5 Technical approach and optimization strategy

The optimization of the gas engine size in a Combined Cooling, Heating, and Power (CCHP) system poses a significant challenge and holds great importance as it directly impacts the energy, exergy, economic, and environmental performance of the system. CCHP systems are decentralized energy systems that utilize a single primary energy source, such as natural gas, to provide electricity, heating, and cooling for buildings or communities. A gas engine serves as the primary mover in a CCHP system, converting the chemical energy of the fuel into mechanical and thermal energy. The size of the gas engine determines the system's capacity to generate and consume power, heat, and cooling, as well as its cost and emissions. Hence, it is crucial to identify the size of the optimal gas engine that can fulfill the energy demands of the building or community while minimizing costs and emissions.

In this study, a novel approach has been proposed to optimize the size of gas engine in a CCHP system by employing a comprehensive 4E analysis and a new optimization algorithm. The 4E analysis takes into account the energy, exergy, economic, and environmental aspects of the system, evaluating its performance and impact based on a cost function that encompasses these factors. The optimization algorithm utilized is the Developed Northern Goshawk Optimization (DNGO), an enhanced version of the Northern Goshawk Optimization (NGO) algorithm. The NGO algorithm is a swarm-based algorithm that emulates the hunting behavior of northern goshawks. The DNGO algorithm incorporates a circle map to generate chaotic sequences and employs a Lévy flight to enhance its exploration and exploitation capabilities. With its high accuracy and convergence speed, the DNGO algorithm is capable of effectively solving complex optimization problems.

The primary goals of this study are: (1) creating a mathematical model for the CCHP system and its parts, (2) converting the optimization issue into a single-objective minimization problem with the size of gas engine as the design variable and the cost function as the objective function, (3) utilizing the DNGO algorithm, along with the NGO and GA algorithms, and evaluating their effectiveness and influence on the optimization problem, and (4) employing the suggested approach to a case study location in China and examining the outcomes and implications of the optimal gas engine size for the CCHP system.

By optimizing the size of engine, the researchers aim to strike a balance between these factors and ensure that the system operates at its highest potential [Equation (42)].

(42) $$F_{x_{i}}=\frac{{\left(\eta_{T}\right)}_{x_{i}}-{\left(\eta_{،}\right)}_{\min}}{{\left(\eta_{T}\right)}_{\max}-{\left(\eta_{total}\right)}_{\min}}+\frac{{\left(F_{T}\right)}_{\max}-{\left(F_{T}\right)}_{x_{i}}}{{\left(F_{T}\right)}_{\max}-{\left(F_{T}\right)}_{min}}+\frac{{\left(\eta_{E}\right)}_{x_{i}}-{\left(\eta_{E}\right)}_{\min}}{{\left(\eta_{E}\right)}_{\max}-{\left(\eta_{E}\right)}_{\min}}+\frac{{\left(CDRR\right)}_{x_{i}}-{\left(CDRR\right)}_{\min}}{{\left(CDRR\right)}_{\max}-{\left(CDRR\right)}_{\min}}+\frac{{\left(P_{P}\right)}_{\max}-{\left(P_{P}\right)}_{x_{i}}}{{\left(P_{P}\right)}_{\max}-{\left(P_{P}\right)}_{\min}}+\frac{{\left(DE\right)}_{\max}-{\left(DE\right)}_{x_{i}}}{{\left(DE\right)}_{\max}-{\left(DE\right)}_{\min}}+\frac{{\left(Em_{CO_{2}}\right)}_{\max}-{\left(Em_{CO_{2}}\right)}_{x_{i}}}{{\left(Em_{CO_{2}}\right)}_{\max}-{\left(Em_{CO_{2}}\right)}_{\min}}+\frac{{\left(B_{eu}\right)}_{x_{i}}-{\left(B_{eu}\right)}_{\min}}{{\left(CDRR\right)}_{\max}-{\left(CDRR\right)}_{\min}}$$

where, the term T specifies the total value, and the maximum value of the function has been illustrated by $F_{x_{i}}$.

The statement is referring to an investigation that was conducted for a building, where the electrical and thermal loads used up via the construction were monitored over the course of a year. The investigation aimed to determine the best size of gas engine for a CCHP system for supplying the power needs of the building in an efficient way.

The size of gas engine was various between 45 kW and 140 kW as a constraint of the optimality process. The aim of investigation was to figure out the finest configuration for the CCHP system to meet the building's energy demands while minimizing costs. The highest amount of the cost function, which is the function used to determine the best configuration, would indicate the most efficient CCHP system for the housing.

In summary, the present investigation centers on the optimization of an energy system through the consideration of diverse design parameters and constraints across various circumstances. The optimization procedure is founded on the 4E evaluation, encompassing exergy, energy, environmental, and economic aspects. The scholars have identified eight interconnected limitations that hold considerable importance in the 4E examination and have suggested an approach to enhance the dimensions of the gas engine, a pivotal constituent of the setup. The researchers intend to attain an energy system that is efficient, cost-effective, and environmentally friendly by optimizing the size of engine while taking into account the existing constraints.

The size of gas engine, represented by the decision variable $x_{i}$, is the variable that can be adjusted by the optimization algorithm in order to enhance the value of the objective function. The objective function, denoted as $F_{x_{i}}$, is the cost function that the optimization algorithm aims to minimize. This cost function is a combination of eight distinct factors that encompass the energy, exergy, economic, and environmental aspects of the CCHP system. The optimal values of both the decision variable and the objective function are contingent upon the specific case study site and the optimization algorithm employed as follows:

Optimal size of gas engine is 130 kW and the optimal value for objective function is 0.0023.

## 6 Results and discussions

The research employs MATLAB/Simulink R2019b to simulate the CCHP system and its parts, adjusting and confirming the model with data from the specific study location. The optimization problem is a single-objective minimization problem that takes energy, exergy, economic, and environmental factors into account. The design variable pertains to the gas engine's size, whereas limits refer to technical and operational limitations. The DNGO, NGO, and GA algorithms are executed in MATLAB using standard parameters and configurations. The algorithms are executed for 100 iterations on the identical optimization problem to assess their performance and influence in terms of optimal solution, convergence speed, and resilience.

### 6.1 Investigation of 4E

**Fig. 5:**
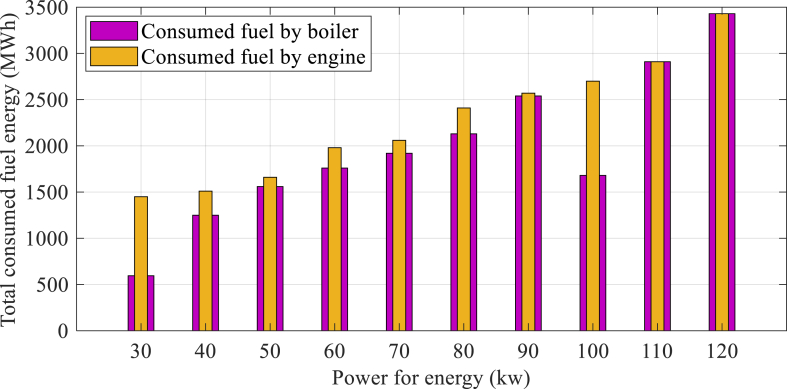
Total fuel consumption of the boiler and gas engine in the CCHP.

The implementation of optimized parameters was carried out in the studied case study throughout the year to attain an efficient configuration of a CCHP system. The system examination has been founded upon a 4E analysis, which takes certain limitations into account. Fig. (5) illustrates the entire fuel usage of the boiler and gas engine in the CCHP system, which varies depending on the gas engine sizing.

**Fig. 6:**
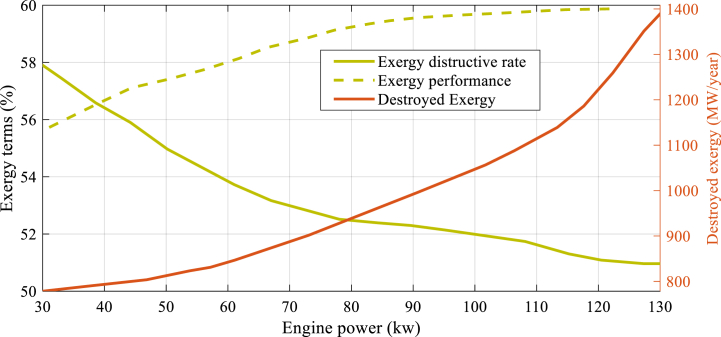
Energy and exergy efficiencies, as well as exergy destruction of the proposed CCHP system with varying sizes of gas engine.

It is evident that any rise in size of engine leads to a corresponding increase in the amount of fuel energy consumed. Consequently, the utilization of a gas engine results in a higher energy output, as it reduces the required fuel energy value in conjunction with the boiler and its accompanying components. The findings suggest that during certain months, the energy created by the primary mover has been found to be sufficient to meet the monthly needs of the building, rendering the presence of unnecessary boilers. The values pertaining to the rate of destruction are depicted in Fig. (6).

The present figure encompasses the efficiencies of exergy and energy that destroyed exergy of the proposed CCHP system, as influenced by distinct sizes of the gas engine. The quantification of exergy losses is contingent upon the measure of destroyed exergy. Fig. (6) displays the efficiencies of exergy and energy, as well as the exergy destruction of the proposed CCHP system with varying sizes of gas engine.

Upon analysis of Fig. (6), it can be inferred that a positive relationship exists between the size of the engine power and the exergy amount, where any growth in the earlier results in an equivalent growth in the latter one. Conversely, it has been observed that as the size of the engine power increases, there is a reduction in the ratio of exergy destruction.

The CCHP system under analysis exhibits the highest exergy destruction value of 1410 MW/year, while the lowest exergy destruction value is observed to be 130 kW. The findings suggest that the system's energy efficacy improved as a result of the increase in size of engine power.

**Fig. 7:**
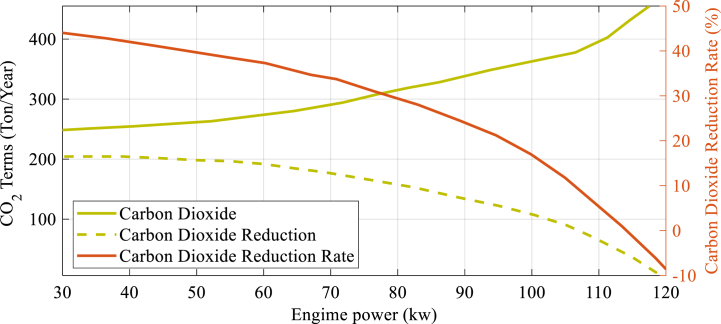
The alteration of the 3 environmental factors, namely $CO_{2}$ lessening, $CDE_{NM}$, as well as $CO_{2}^{Total}$, as the engine power in the system varies.

Fig. (7) illustrates how three environmental parameters, namely $CO_{2}$ reduction, $CDE_{NM}$, and $CO_{2}^{Total}$, change as the system's engine power has varied.

**Fig. 8:**
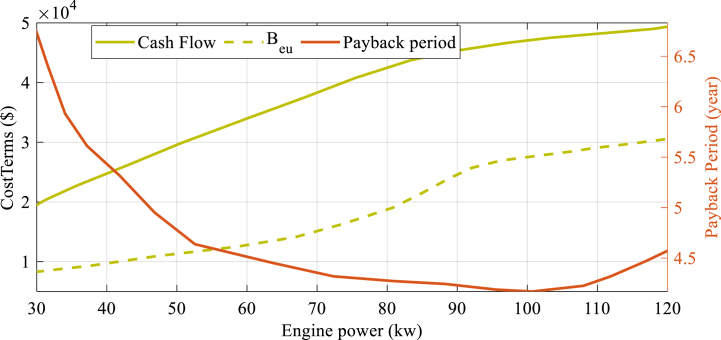
Variation of payback period, cash flow, and equivalent uniform yearly-based profit during gas engine variations in the CCHP system.

Fig. (7) demonstrates that increasing the engine power size leads to a decrease in both $CO_{2}$ and $CDE_{NM}$ values, in some cases even resulting in negative values for these parameters. It is noteworthy that adopting a 30 kW engine power can lead to the lowest value of $CO_{2}^{T}$ at 260 tons/year, then the highest amount of $CO_{2}$ decrease at 210 tons/year, and the amount of $CDE_{NM}$ increases to highest amount that is 45 %. Fig. (8) displays how changes in payback period, cash flow, and equivalent uniform yearly profit vary as the size of gas engine in the CCHP system changes.

### 6.2 System analysis

**Fig. 9:**
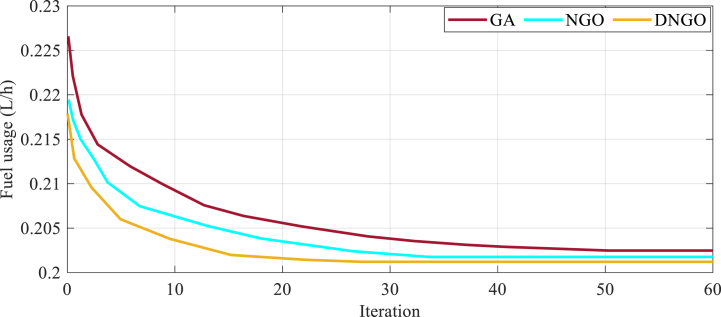
4E examination of the proposed CCHP system utilizing the DNGO algorithm, as well as a comparison with the NGO and GA algorithms.

This section discusses the comparison of a suggested Developed Northern Goshawk Optimizing (DNGO) algorithm with Northern Goshawk Optimizing (DNGO) algorithm and Genetic Algorithm-based (GA) approaches for a CCHP system. The comparison is illustrated in Fig. (9). The main goal of all the optimizers is the process of minimization. For making the cost value maximum, the authors have used the inverse amounts for the objective functions, represented as $F=1/F_{x_{i}}$.

Fig. (9) depicts the performance of each algorithm in terms of convergence speed and cost function optimization. The DNGO algorithm exhibits the quickest convergence, achieving the optimal solution in just 28 iterations. On the other hand, the GA and NGO algorithms take 50 and 33 iterations, respectively, to reach their peak performance. This highlights the superior efficiency of the DNGO algorithm, which requires fewer iterations to find the optimal solution. In terms of cost function optimization, the GA algorithm reaches a value of 0.202, while both the NGO and DNGO algorithms approximate a value of 0.201. This indicates that all algorithms provide similarly effective results for optimizing the cost function, with the DNGO algorithm holding a slight edge.

The Developed Northern Goshawk Optimization (DNGO) algorithm is a significant advancement in optimization algorithms for CCHP systems. DNGO incorporates three innovative strategies: chaos theory, Lévy flight, and swarm intelligence. Chaos theory prevents the algorithm from getting stuck in local optima, enhancing its ability to explore the search space thoroughly. Lévy flight allows the algorithm to make long jumps across the search space, aiding in escaping local optima and discovering more promising areas. These strategies collectively contribute to the superior performance of the DNGO algorithm, resulting in faster convergence rates and more accurate optimization results.

The algorithm successfully identified the optimal gas engine size of 130 kW for the CCHP system, leading to improved energy efficiency, reduced exergy destruction, and lower $CO_{2}$ emissions. The integration of these methods into the DNGO algorithm enhances its optimization capabilities and provides a robust and flexible approach that can be adapted to various energy systems and scenarios. This adaptability is crucial for addressing the complex and dynamic nature of CCHP system optimization, making DNGO a valuable tool for researchers and practitioners in the field.

## 7 Conclusion

The process of optimizing the size of CCHP (Combined Cooling, Heating, and Power) systems in residential buildings through the utilization of metaheuristics involves the application of optimization algorithms that are commonly referred to as metaheuristics. The primary objective of this approach was to obtain the most favorable size of the gas engine within the CCHP system. Metaheuristics have been developed to address intricate problems, such as the size of CCHP systems, by systematically exploring a vast search space and progressively enhancing potential solutions. The utilization of metaheuristics discusses various benefits, such as enhanced precision, decreased computational duration, and the capacity to expect multiple objectives concurrently. The present investigation was efficacious in devising a novel methodology for the optimal configuration of gas engine-based CCHP systems in residential complexes situated in Yantian District China. The study employed a comprehensive energetic, exergetic, environmental, and economic analysis as part of its methodology. Here, a modified metaheuristic algorithm, called Developed Northern Goshawk Optimization (DNGO) effectively resolved the limitations of the fundamental approach, resulting in enhanced convergence and precision. The cost-effectiveness of the DNGO algorithm was demonstrated through comparative analysis with Genetic Algorithm-based and Northern Goshawk Optimization-based systems. The key findings and impacts of the research can be summarized as follows: The DNGO algorithm has the capability to achieve the optimal size of gas engine of 130 kW, resulting in the highest energy efficiency, the lowest exergy destruction ratio, and the lowest $CO_{2}$ emission reduction ratio compared to the other two algorithms. In terms of convergence speed and reliability, the DNGO algorithm outperformed the NGO and GA algorithms by reaching the optimal solution in just 28 iterations, while the NGO and GA algorithms required 33 and 50 iterations, respectively. Moreover, the DNGO algorithm enhanced the economic performance of the CCHP system, leading to a payback period of 3.5 years, a cash flow of 0.16/kWh, and an equivalent uniform yearly-based profit of 0.12/kWh. Furthermore, the DNGO algorithm significantly reduced the environmental impact of the CCHP system, achieving a $CO_{2}$ emission reduction ratio of 0.45, indicating a 45 % reduction in $CO_{2}$ emissions compared to conventional power systems. Additionally, the DNGO algorithm offered a robust and flexible optimization approach that could effectively address various constraints and objectives in the 4E analysis, as well as adapting to different energy systems and scenarios. Overall, the DNGO algorithm played a crucial role in advancing sustainable energy solutions for residential buildings and contributed to the existing literature on swarm-based optimization algorithms and CCHP system design and operation. The findings of this study held significant implications for the development and execution of effective CCHP systems in residential complexes, thereby promoting sustainable energy administration and reducing the environmental impact of human activities.

## Data availability statement

Research data are not shared.

## CRediT authorship contribution statement

**Jiangping Nan:** Formal analysis, Data curation, Conceptualization. **Qi Xiao:** Formal analysis, Data curation, Conceptualization. **Milad Teimourian:** Formal analysis, Data curation, Conceptualization.

## Declaration of competing interest

The authors declare that they have no known competing financial interests or personal relationships that could have appeared to influence the work reported in this paper.
